# Feasibility of Combining Functional Mobilisation with Resistance and Endurance Training for Mechanically Ventilated Patients in Intensive Care Unit Setting—A Pilot Study

**DOI:** 10.3390/jcm13082412

**Published:** 2024-04-20

**Authors:** Balachandran Jayachandran, Kumaresh Venkatesan, Sunny Boon Chai Tan, Lynn Soo Hoon Yeo, Jonathen Venkatacham, Mohena Priyaa Selvakumar, Bryan Yijia Tan

**Affiliations:** 1 Rehabilitation Department, Woodlands Health, 17 Woodlands Dr 17, Singapore 737628, Singapore; mohena_priyaa_selvakumar@wh.com.sg; 2 Anaesthesia Department, Khoo Teck Puat Hospital, 90 Yishun Central, Singapore 768828, Singapore; 3 Rehabilitation Department, Khoo Teck Puat Hospital, 90 Yishun Central, Singapore 768828, Singapore; tanboonchaisunny@gmail.com; 4 Anaesthesia Department, Woodlands Health, 17 Woodlands Dr 17, Singapore 737628, Singapore; lynn_yeo@wh.com.sg; 5 General Medicine, Khoo Teck Puat Hospital, 90 Yishun Central, Singapore 768828, Singapore; jonathen.venkatachalam@ktph.com.sg; 6 Orthopaedic Surgery, Woodlands Health, 17 Woodlands Dr 17, Singapore 737628, Singapore; bryan_tan@wh.com.sg

**Keywords:** functional training, resistance training, endurance training, functional outcome, mechanical ventilation

## Abstract

**Background:** Intensive-care-acquired weakness resulting in functional impairment is common in critical care survivors. This study aims to evaluate the feasibility of a combined early functional training with endurance and resistance training and its effect on the functional outcome. **Methods:** It is a pilot study performed in a 39-bed Medical and Surgical Intensive Care Unit (ICU). Patients who were premorbidly independent and were mechanically ventilated for ≥24 h were recruited to receive functional mobilisation (sit out of bed, ambulation), endurance (bed cycling), and resistance training (selected upper and lower limb muscle training using weights). The primary outcomes were feasibility of training, muscle strength, handgrip strength, quadricep strength, and Functional Status Score-Intensive Care Unit (FSS-ICU) collected at the first assessment in the ICU, at the ICU discharge, and at hospital discharge. Secondary outcomes were functional capacity (6-Minute Walk Distance) and quality of life measures, EQ-5D, at hospital discharge and at 3 months. **Results:** Out of the 11 patients, 6 (54.54%) patients achieved level 2 functional mobilisation, 2 (18.18%) patients achieved level 2 resistance training, and 1 (9.09%) patient achieved level 2 endurance training. There were no significant differences in the medical research council (MRC) score, quadricep strength, and handgrip strength between the first assessment in the ICU, at the ICU discharge, and at hospital discharge. However, there was a significant difference in FSS_ICU (*p* < 0.008) from the first assessment in the ICU up to hospital discharge. EQ-5D visual analogue scale also showed a change of 8.5% at 3-month follow-up. 6MWD showed significant difference (*p* < 0.043) at 3-month follow-up compared to that at hospital discharge. **Conclusions:** The study found low compliance to resistance and endurance training in patients with mechanical ventilation. However, functional mobilisation in terms of sit out of bed was possible in more than half of the recruited patients.

## 1. Introduction

Muscle weakness, myopathy, muscle atrophy, and critical illness neuropathy are common in critically ill patients, with up to 80% of patients developing some form of neuromuscular dysfunction [1]. Symptoms of intensive-care-acquired weakness (ICUAW) develop within the first week of critical illness [2]. Its risk factors include sepsis, multiple organ failure, mechanical ventilation, immobilisation, hyperglycaemia, and use of vasoactive medications [3,4]. The short-term consequences of ICUAW with limb and respiratory muscle weaknesses have been identified as independent predictors of prolonged need of mechanical ventilation [5,6] and high extubation failure rates in medical patients [7]. The durations of ICU and hospital stays are longer, with increased in-hospital costs [8,9]. In addition, ICUAW is associated with the substantial impairment in physical function [10] and health-related quality of life (HRQOL) that persist at 24 months [11].

Active mobilisation and rehabilitation in the ICU have shown to improve mobility status and muscle strength [12], with significant functional benefits, which may translate into a reduced ICU and hospital LOS [13]. Cycle ergometry is a potentially safe and feasible strategy for ICU-based rehabilitation, currently with less evidence to improve physical function on its own [14]. However, when cycle ergometer was combined with resistance training, it has shown to improve lower limb muscle strength, walking ability, and cardiorespiratory fitness during the inpatient rehabilitation of intensive-care-acquired weakness [15]. The efficacy of combining mobilisation with cycle ergometer and resistance training in an ICU setting and its effect on ICUAW have not been widely studied and evaluated.

The aim of this pilot study is to evaluate the feasibility of a novel combined early functional mobilisation, endurance training with cycle ergometer, and resistance training in mechanically ventilated ICU patients. The secondary aim is to investigate the influence of the combined training program on the functional outcomes of patients.

## 2. Materials and Methods

### 2.1. Design and Setting

This is a pilot study performed in a 39-bed Medical and Surgical ICU in a regional hospital in Singapore. The study was conducted from 1 December 2022 to 30 June 2023. The study was approved by an ethics committee (Domain Specific Review Board Reference Number: 2020/01186). The written informed consent was taken from recruited patients directly.

### 2.2. Participants

The inclusion criteria of the study are as follows: 1. adults (21 years and above) expected to receive invasive mechanical ventilation for ≥24 h, 2. able to ambulate independently before hospital admission (with or without a gait aid), 3. admission to the ICU due to respiratory infection, respiratory failure, sepsis, post abdominal surgery or other surgeries, and procedures requiring intubation for ≥24 h, and 4. patients able to follow three out of the five commands, i.e., a. open/close your eyes, b. look at me, c. open your mouth and stick out your tongue, d. nod your head, and e. raise your eyebrow when I have counted to five [16]. The exclusion criteria of the study are as follows: 1. patients with new neurological insult (e.g., stroke) or any loss of body parts (upper limb or lower limb), 2. fractures, acute thrombosis, graft, or flap surgeries to lower limbs, 3. bodyweight more than 135 kg, 4. patient on palliative care, 5. unable to follow commands, and 6. readmission to the ICU during the current admission.

### 2.3. Procedures

Patients were screened daily for eligibility by one of the study team members. Once they met the eligibility criteria, patients were reviewed and deemed clinically appropriate by the medical team for inclusion in the study. Consent was obtained by one of the study team members in the presence of an impartial witness (nurses, physiotherapist, and physiotherapy assistant), who was not part of the study team. After patient consented to the study, interventions were delivered as per patient’s tolerance. The first assessment in the ICU was carried out by the study team physiotherapist. The subsequent assessments at the ICU discharge, hospital discharge, and 3-month follow-up were performed by an independent physiotherapist who was not a part of the study team. At the time of hospital discharge, outpatient appointment was pre-scheduled for the 3-month follow-up. Appointment reminder was sent to the patient 1 day prior to the scheduled appointment through an automated short message service to the individuals registered mobile devices and followed up a phone call reminder by the study team member.

On the day of appointment, when patient arrived in the outpatient department, patient’s vital signs were checked, and EQ-5D and 6MWD were assessed by the outpatient physiotherapist (not a part of the study team) and updated in the electronic medical records.

### 2.4. Training Protocol

All recruited patients were screened for contraindications (Table S1 Contraindications) for different systems (cardiovascular and respiratory) and laboratory investigation before intervention as per hospital guidelines. The interventions included, functional mobilisation, endurance, and resistance training (Table 1). The sessions were delivered in blocks of approximately 20 min during daytime to avoid overexertion. The interventions were carried out during weekdays only till the patient was discharged from the ICU. Functional mobilisation consists of sit over edge of bed (SOEOB), sit out of bed (SOOB), and sit to stand and ambulate as tolerated. Patients were considered to achieve training protocol if they managed to achieve sit out of bed (level 2). Endurance training was conducted with motor-assisted bed cycle (MOTOmed viva2, Reck-Technik, Betzenweiler, Germany) that allows passive, motor-assisted, or active cycling in bed. The ergometer had the capability to transition from passive to active mode at a fixed pedalling rate of 20 cycles/min depending on patients’ participation. Endurance training protocol was adapted from a previous study performed by Burtin et al. in 2009 [17]. Patients were considered to achieve training protocol if they managed to achieve 20 min of active endurance training (level 2). Resistance training was conducted for a selected group of upper (deltoid and biceps) and lower limb (psoas major and quadriceps) muscles using weights or dumbbells. The training intensity was set to 8–12 repetitions of 1–2 sets with 50–70% of measured one-repetition maximum. In patients who were unable to perform resistance exercises because of lack of strength, active or active-assisted exercise without weights were delivered. Resistance training protocol was adapted from a previous study performed by Eggman et al. in 2018 [18]. Patients were considered to achieve training protocol if patient managed to achieve resistance training at 50–70% of one repetition maximum for at least one set of 8–12 repetitions.

Upon discharge from the ICU, further interventions were delivered by a physiotherapist in the general ward based on their current assessment.

### 2.5. Staff Training

All study team members and the independent physiotherapist involved in delivering the intervention and conducting outcome measurement were trained using simulated patient, and their competency was checked. This was to ensure the reliability of the outcome measures assessed at different time points.

### 2.6. Monitoring and Criteria for Interruption

During interventions, patient’s blood pressure, heart rate, oxygen saturation, and respiratory rate were continuously monitored. Subjectively, patients were monitored for chest pain, shortness of breath, and headache before, during, and after intervention. During the sessions, interventions were discontinued if any of the criteria for interruption (Table 2) was met.

### 2.7. Measurements

Baseline characteristics such as patient’s age, comorbidities, premorbid functional status, ventilation days, vasopressor support, continuous renal replacement therapy (CRRT), and the ICU admission diagnosis were collected. Primary outcomes collected were the following: 1. Feasibility (compliance) of training protocol. Training protocol compliance, defined as achieving level 2 of functional mobilisation (sit out of bed), level 2 of resistance training (50–70% of one repetition maximum, one set of 8–12 repetitions), and level 2 of endurance training (20 min (2 × 10 min)) of active cycling, was measured. Along with the protocol compliance, training volume of each intervention was collected. Other primary outcomes collected were as follows: 2. Manual muscle strength assessed using the medical research council (MRC) score for three bilateral muscle groups in upper extremities (shoulder flexors, elbow flexors, and wrist extensors) and lower limbs (hip flexors, knee extensors, and ankle dorsiflexors). Manual muscle testing scoring was measured using six-point medical research council scale read from 0 (no palpable muscle contraction) to 5 (normal muscle strength). 3. Handgrip strength was measured using Jamar hydraulic hand dynamometer (Performance Health International LTD, Huthwaite, UK) 4. Quadricep strength was measured using handheld dynamometer (microFET 2, Hoggan Scientific, LLC, Salt Lake City, UT, USA). 5. Functional status was assessed using Functional Status Score-Intensive Care Unit (FSS-ICU) [19]. Measurements were collected at baseline (the first ICU assessment), at the ICU discharge, and at hospital discharge. Manual muscle strength, handgrip strength, and quadricep strength assessments were carried out twice, and the best results were considered for analysis. Secondary outcomes collected were functional capacity (6-Minute Walk Distance (6MWD) [20] and quality of life measures, EQ-5D. Measurements were collected at hospital discharge and at 3-month follow-up after hospital discharge. Adverse events (dislodgement of lines, accidental falls, and hemodynamic instability) that occurred during intervention or up to 15 min after physiotherapy and persisted despite an intervention or therapy interruption were also collected.

### 2.8. Statistical Analysis

Data were explored and analysed using SPSS version 27.0. Descriptive statistics were used to describe the characteristics of patients. Continuous data were presented as median (25th percentile, 75th percentile), while categorical variables were presented as frequency and percentage. The changes in primary outcomes between the first assessment, the ICU discharge, and hospital discharge were explored using the Wilcoxon signed rank test. All the tests were two sided, and the statistical significance was denoted by *p* < 0.05.

## 3. Results

### 3.1. Study Participants

**Figure 1:**
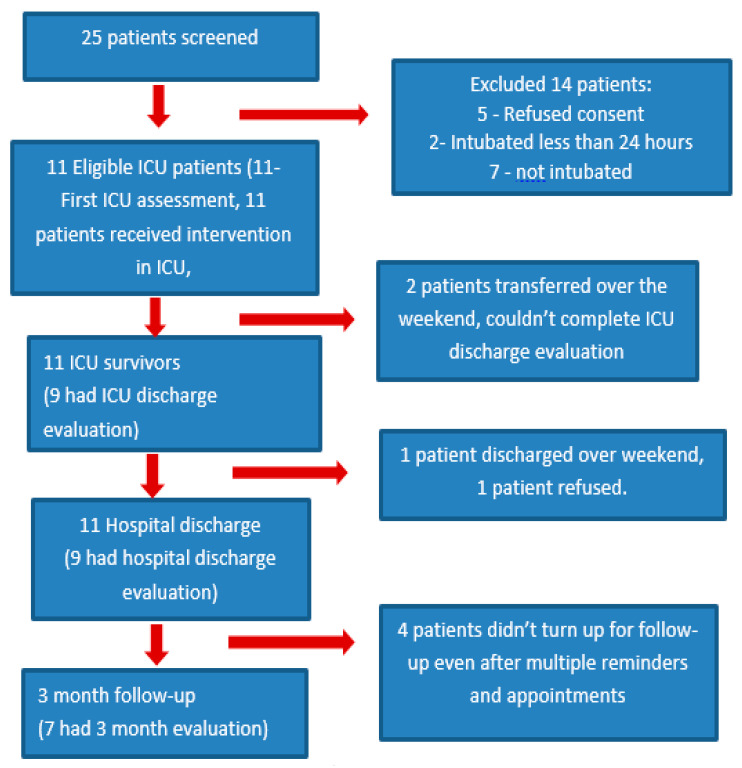
Study flow diagram.

A total of 25 SICU and MICU patients were screened, and 11 patients were recruited based on the study criteria over a period of 7 months from December 2022 to June 2023 (Figure 1). Patient demographics are described in Table 3. The median time from ICU admission to recruitment was 3 (IQR 2.5, 4) days, and the median time from ICU admission to intervention was 3 (IQR 2.5, 4) days. Patient’s length of stay in ICU was a median of 5 days (IQR 4, 7.5). Patient’s length of stay in hospital was a median of 16 days (IQR 10, 21.5).

### 3.2. Training Protocol Compliance

Out of the 11 patients, 6 (54.54%) patients achieved level 2 functional mobilisation, 2 (18.18%) patients achieved level 2 resistance training, and 1 (9.09%) patient achieved level 2 endurance training (Table 4). The main reason for decreased compliance to study intervention especially resistance and endurance training were patient’s refusal due to fatigue, the presence of femoral catheter, ongoing CRRT, and short stay in the ICU.

### 3.3. Training Volume

Physiotherapy intervention started immediately after patients consent on day 3 of the ICU admission. There were a total of 56 sessions of functional mobilisation. Most of the patients received a median of 1.5 sessions (1, 2) of sit over edge of bed activities. For resistance training, there were a total of 21 sessions, and almost all patients received a median of one session (IQR 1, 2) of active range of motion exercise without weights. For resistance training with weights, only two patients received two sessions of training. For endurance training, only one patient received two session of endurance training with cycle ergometer (Table 5).

### 3.4. Adverse Events

There were no adverse events reported with a total of 56 sessions of functional mobilisation, 20 session of resistance training, and 2 sessions of endurance training.

### 3.5. Primary Outcomes

There were no significant differences in the MRC score, quadricep strength, and handgrip strength from first assessment in the ICU to hospital discharge. There was no significant difference between FSS_ICU from the first assessment in the ICU to the ICU discharge; however, there was a significant difference in FSS_ICU (*p* < 0.008) from the first assessment in the ICU to hospital discharge (Table 6).

### 3.6. Secondary Outcome

EQ-5D showed an improvement at the 3-month follow-up compared to that at hospital discharge. Overall health that was assessed using a visual analogue scale also showed a change of 8.5% at the 3-month follow-up. 6MWD showed a significant difference at the 3-month follow-up compared to that at hospital discharge (Table 7).

## 4. Discussion

Most of the studies have explored the feasibility and impact of daily functional mobilisation in critically ill patients in the ICU. The purpose of our pilot study was to explore the feasibility of combining daily functional mobilisation with daily resistance and daily endurance training and its impact on the ICU patients who were mechanically ventilated for more than 24 h. We found that, while we were able to deliver daily functional mobilisation in 50% of patients, we achieved lower compliance in daily resistance (18%) and endurance training (9%). The main reasons for the decreased compliance to resistance and endurance training were fatigue and the refusal to participate by patients (45.45%). The other reasons were the presence of femoral vascular catheters (18.18%), patients undergoing CRRT (18.18%), and patients discharged to the GW from the ICU (18.18%). The low compliance to resistance training was also reported by an earlier study (Eggmann et al., 2018) [17], which combined early mobilisation and resistance and endurance training. In that study, there were only 8 resistance training sessions out of a total of 407 physiotherapy sessions. The authors attributed the low compliance to resistance training and to the shorter length of stay in the ICU (median: 6 days). Another study (Sue Berney et al., 2012) [21], which prescribed endurance, resistance, and functional mobility starting in the ICU and continuing through acute care and followed-up in outpatients, found that exercise sessions were not delivered 45% (527 sessions) of the time in the ICU. In their study, the main attributed reason for low compliance was patient refusal due to fatigue. In contrast to our study findings, a study (Kimawi et al., 2017) [22] using a protocolised approach for endurance training using a cycle ergometer achieved 96% compliance, However, the study did not study the simultaneous implementation of functional mobilisation and resistance training. The study reported delivery of endurance training even in the presence of femoral catheter and CRRT, which was one of the barriers for endurance training in our study.

Our study managed to achieve a slightly higher compliance to functional mobilisation compared to resistance and endurance training, and one of the reasons for this could be the previous quality improvement project on ICU early mobilisation that had changed the beliefs and attitudes towards early functional mobilisation in the critical care unit.

The combined delivery of functional mobilisation with resistance and endurance training is challenging in our setting based on the findings of our feasibility study. The main attributable reasons could be (1) the shorter length of stay in the ICU (median: 5 days), which gives limited time to implement more than one training protocol, and the other reasons could be (2) the perceptions and beliefs about the importance of physical exercise in our population of patients recovering from critical illness, (3) competing priorities with medical and nursing care, limiting the therapy sessions, and (4) easy fatigability of patient recovering from critical illness, leading to their refusal in participating in therapy session.

Based on our study observation, we feel that the therapy session needs to be more flexible and tailored to the needs and tolerance of patient in the ICU setting. We also observed our patients stayed longer in the general ward (median: 16 days). Therefore, for patients with shorter LOS in the ICU, we should aim to start with functional training in the ICU and introduce resistance and endurance training in the general ward setting. Long stayers in the ICU would warrant a different approach and interventions scheduled to be delivered on different timings on the same day or on different days, to improve the tolerance and compliance to resistance and endurance training.

We did not observe any adverse events like the dislodgement of lines, accidental falls, and hemodynamic instability in our study. One of the reasons for no adverse events in our study may be the stringent safety criteria of excluding patients on vasopressor and inotropic medications from interventions. In contrast to our study, Burtin and colleagues [17] used a ceiling vasopressor dosage, below which an intervention was permitted, and they demonstrated the safe delivery of intervention in an ICU setting. We should take this into consideration for our future practice, so that physiotherapy sessions will not be limited due to stringent safety criteria.

Our study did not find any significant difference in the MRC muscle score, quadricep strength, and hand grip strength at the ICU discharge and at hospital discharge compared to the first ICU assessment. This implies that patient did not develop muscle weakness and did not develop ICUAW (Table 6). The FSS_ICU score was on the lower side at the first ICU assessment (22/35) and the ICU discharge (23/35) and did not show any significant difference at the ICU discharge. The main reason for a low FSS-ICU was the ambulation domain, which was difficult may be due to the effect of sedative medications or mild deconditioning due to bed rest.

However, FSS_ICU continued to improve and showed a significant difference at hospital discharge (*p* < 0.008) compared to FSS_ICU at the first ICU assessment (Table 5). The change in FSS_ICU score at hospital discharge also translated into patients being able to return to premorbid mobility at hospital discharge. This was possible even with the majority of patients only receiving functional mobilisation during their ICU days. Some of the reasons for retaining their premorbid mobility at hospital discharge could be the following: patients did not develop ICUAW, interventions were started as early as day 3 of the ICU admission, and patient profile (a median age of 51 (IQR 42, 72) and premorbidly independent.

Our study also found that patients followed up at 3 months showed a significant improvement (*p* < 0.043) in their exercise capacity measured using the 6-Minute Walk Distance compared to hospital discharge measurement. One of the reasons could be that our functionally independent patients at hospital discharge continued to engage in physical activities at home and community, resulting in an overall improvement in functional capacity and quality of life. The quality of life measured with the EQ-5D also showed improvement in all the five domains (mobility, self-care, usual activities, pain/discomfort, and anxiety/depression) at 3-month follow-up compared to that at hospital discharge (Table 6). Their perception of health assessed using a visual analogue scale showed an improvement of 8.5% at 3-month follow-up compared to that at hospital discharge. Even though most of the patients were independent at hospital discharge and 6MWD showed a significant improvement post discharge, the EQ-5D QOL assessment showed that 38% of the patients had some problems with walking and self-care and 50% had some problem with anxiety. This is a finding and we could not find any association for.

### Limitations and Strength

The sample size is small, and it is difficult to draw conclusions from the findings of our study. Our recruitment was delayed due to the restrictions imposed by the ethics committee. The ethics committee’s restriction prevented surrogate consenting from legally identified next of kin and consent was to be obtained only from patients. This delayed the recruitment of patients and limited the amount of time and interventions that patients could receive prior to their discharge from the ICU once they were medically stable. Despite limitations, the study provided us with insights into the potential barriers to resistance and endurance training. It also helped us to understand the local practice challenges compared to other practices across the world.

## 5. Conclusions

This pilot study found low compliance to resistance and endurance exercise training in mechanically ventilated patients. Functional mobilisation was possible in more than half of the study patients. Majority of the patients were able to regain their functional status at hospital discharge with improvement in 6-Minute Walk Distance at the 3-month follow-up.

## Figures and Tables

**Table 1:** Training protocol.

Functional Mobilisation	Resistance Training	Endurance Training
Level 1—sit over edge of bed (SOEOB)	Level 1—active/active-assisted exercise	Level 1—passive cycling, 20 min, 20 cycles/min.
Level 2—sit out of bed (SOOB)	Level 2—resistance exercise at 50–70% (one repetition maximum) 1 set of 8–12 reps	Level 2—active cycling 20 min (2 × 10 min), 20 cycles/min
Level 3—sit to stand (STS)	Level 3—resistance exercise at 50–70% (one repetition maximum) two sets of 8–12 reps	Level 3—active cycling 30 min (2 × 15 min), 20 cycles/min *Resistance is increased as tolerated from 0 to 6 only after reaching 30 min as a whole*
Level 4—ambulate		

**Table 2:** Criteria for interruption.

1	Blood pressure: mean arterial pressure < 65 mmHg or mean arterial pressure > 110 mmHg
2	Heart rate: <40 or >140 beats per minute
3	Spo2 <88% or drop by 3% from baseline
4	Respiratory rate: >24 breaths per minute
5	Suspicion of cardiac ischemia or new arrhythmias
6	Drop in the Glasgow Coma Scale or sudden patient agitation
7	Subjective symptoms (chest pain, shortness of breath, or patient’s request to stop)

**Table 3:** Patient demographics.

Age in years, median (IQR)	51 (42–72)
• Gender, n (%)	
• Male	6 (54.54)
• Female	5 (45.45)
Race, n (%)	
1. Chinese	3 (27.3)
2. Malay	4 (36.4)
3. Indian	4 (36.4)
Co-morbidities, n (%)	
1. Ischemic heart disease	2 (18.18)
2. Chronic heart failure	1 (9.09)
3. Hypertension	6 (54.54)
4. Diabetes mellitus	4 (36.36)
5. Hyperlipidaemia	4 (36.36)
6. Chronic obstructive pulmonary disease	4 (36.36)
7. Peripheral vascular disease	1 (9.09)
8. Cerebral vascular accident	0 (0)
Premorbid mobility status, n (%)	
1. Independent without aid	10 (90.90)
2. Independent with aid	1 (9.09)
Activities of daily living (ADL) status, n (%)	
1. Independent without assistance	11 (100)
2. Needs assistance	0 (0)
ICU admission type, n (%)	
1. Emergency department	10 (90.90)
2. General ward	1 (9.09)
ICU admission to recruitment, median (IQR)	3 (2.5–4)
ICU admission to intervention, median (IQR)	3 (2.5–4)
Number of ventilator days, median (IQR)	3 (2.5–4)
Vasopressor support on admission *n* (%)	9 (81.81)
CRRT on admission *n* (%)	3 (27.27)
Diagnosis by subgroup n (%)	
1. Abdominal surgery (exploratory laparotomy for bowel resection, omental patch repair, nephrectomy, and Hartmann’s procedure)	4 (36.36)
2. Type 1 respiratory failure (bilateral pneumonia)	3 (27.27)
3. Type 2 respiratory failure (infective exacerbation of chronic obstructive pulmonary disease, acute pulmonary oedema, and bronchopneumonia)	2 (18.18)
4. Sepsis (leptospirosis and pyelonephritis)	2 (18.18)
ICU length of stay (LOS), median (IQR)	5 (4–7.5)
Hospital length of stay (LOS), median (IQR)	16 (10–21.5)

**Table 4:** Training protocol compliance.

Intervention Protocol	Number of Patients	Reasons for Inability to Achieve Level 2 Protocol Intervention (%)
Functional mobilisation protocol compliance		Presence of femoral catheter (18.18) Ongoing CRRT (18.18) Patient’s refusal (45.45) Discharge from ICU (18.8)
1. Met, n (%)	6 (54.54)	
2. Unmet, n (%)	5 (45.45)	
Resistance training protocol compliance		
1. Met, n (%)	2 (18.18)	
2. Unmet, n (%)	9 (81.81)	
Endurance training protocol compliance		
1. Met, n (%)	1 (9.09)	
2. Unmet, n (%)	10 (90.90)	

**Table 5:** Training volume.

Intervention Protocol	Number of Patients Subjected to Intervention Protocol	Number of Sessions	Session per Patient, Median (IQR)
Functional mobilisation			
1. Sit over edge of bed	10	17	1.5 (1, 2)
2. Sit out of bed	5	12	2 (1, 3)
3. Sit to stand	9	17	1 (1, 2)
4. Ambulate	6	10	1.5 (1, 2)
Resistance training			
1. Without weights	11	17	1 (1, 2)
2. With weights	2	4	2 (2, 2)
Endurance training			
1. Active	1	2	2 (2, 2)
2. Passive	0	0	0

**Table 6:** Primary outcome.

Primary Outcome	No of Patients in Whom Assessment Was Performed at Baseline (First ICU Assessment)	Median (IQR)	Number of Patients in Whom Assessment Was Performed at ICU Discharge	Median (IQR)	*p* Value	Number of Patients in Whom Assessment Was Performed at Hospital Discharge	Median (IQR)	*p* Value
MRC sum-score	11	60 (48.5–60)	7	53 (51.5–58.5)	1.00	9	58 (56–60)	0.223
Quadricep Strength (kg)								
Right	10	11.4 (9.67–13.05)	6	10.9 (7.57–13.7)	0.893	7	13.1 (10.55–14.2)	0.176
Left	11	11.7 (8.95–13.1)	6	9.4 (8.62–10.17)	0.173	7	11.3 (10.4–13.3)	0.866
Handgrip Strength (kg)								
Right	11	18 (10–21)	7	13 (8.5–16)	0.686	9	18 (14–23)	0.499
Left	11	18 (9–22)	7	12 (10.5–20)	0.865	9	14 (12–23)	0.235
FSS_ICU	10	22 (18.5–22.75)	9	23 (19–25.5)	0.273	9	35 (28–35)	0.008

**Table 7:** Secondary outcome.

Secondary Outcome	At Hospital Discharge *n* = 8	At 3-Month Follow-Up *n* = 8	*p* Value
Mobility n (%)			
1. I have no problems in walking about	5(62.5)	5 (62.5)	
2. I have slight problems in walking about	1(12.5)	2 (25)	
3. I have moderate problems in walking about	1 (12.5)	1 (12.5)	
4. I have severe problems in walking about	1 (12.5)	0 (0)	
5. I am unable to walk about		0 (0)	
Self-Care n (%)			
1. I have no problems washing or dressing myself	0 (0)	8(100)	
2. I have slight problems washing or dressing myself	5 (62.5)	0(0)	
3. I have moderate problems washing or dressing myself	1 (12.5)	0 (0)	
4. I have severe problems washing or dressing myself	2 (25)	0 (0)	
5. I am unable to wash or dress myself	0 (0)	0 (0)	
Usual Activities n (%)			
1. I have no problems doing my usual activities	4(57.14)	6 (75)	
2. I have slight problems doing my usual activities	2(28.57)	2 (25)	
3. I have moderate problems doing my usual activities	1(14.28)	0 (0)	
4. I have severe problems doing my usual activities	0(0)	0 (0)	
5. I am unable to do my usual activities	0 (0)	0 (0)	
Pain/Discomfort n (%)			
1. I have no pain or discomfort	4 (50)	4(50)	
2. I have slight pain or discomfort	3 (37.5)	3(37.5)	
3. I have moderate pain or discomfort	1 (12.5)	1(12.5)	
4. I have severe pain of discomfort	0 (0)	0 (0)	
5. I have extreme pain or discomfort	0 (0)	0(0)	
Anxiety/Depression n (%)			
1. I am not anxious or depressed	4(50)	5(62.5)	
2. I am slightly anxious or depressed	3(37.5)	1(12.5)	
3. I am moderately anxious or depressed	1(12.5)	2(25)	
4. I am severely anxious or depressed	0 (0)	0 (0)	
5. I am extremely anxious or depressed	0(0)	0(0)	
Your Health Today			
Visual analogue scale (0–100), median (IQR)	69 (52.5-75)	77.5 (68.75-86.25)	
Functional Capacity			
6-Minute Walk Distance, median (IQR)	181.5 (44-225.5)	320 (285.35-427.35)	0.043

## Data Availability

Data are unavailable due to ethical restrictions.

## Supplementary Materials

The following supporting information can be downloaded at: https://www.mdpi.com/article/10.3390/jcm13082412/s1, Table S1: Table Contraindications S1.
