# Supplementary material for: Feasibility of Combining Functional Mobilisation with Resistance and Endurance Training for Mechanically Ventilated Patients in Intensive Care Unit Setting—A Pilot Study
Source: J Clin Med. 2024 Apr 20;13(8):2412. doi: 10.3390/jcm13082412 (PMC11051037; doi:10.3390/jcm13082412)
Supplement: Supplementary file 1 [file jcm-13-02412-s001.zip › jcm-2931252-supplementary.pdf]

Journal of
Clinical Medicine

Supplementary Materials

Table S1. Table Contraindications S1.

**Check for contraindication:**
**Cardiovascular**
• Heart rate>100bpm or Heart rate change of 20 beats from baseline
• Hypotension, MAP < 65 or Hypertension, MAP > 110mmhg–
• On inotropes support (noradrenaline > 0.05mcg/kg/minute, vasopressin > 1.2units/hour dopamine > 4mcg/kg/minute or more than one inotrope)
• Abnormal ECG (e.g., presence of unstable arrhythmias or myocardial infarction) in the last 24 hours)
• Intravenous Glyceryl Trinitrate/Labetalol
**Respiratory system**
• Spo2 < 92%
• Pao2/ Fio2 ratio < 200
• Respiratory Rate > 30 breaths per minute
• PEEP>10
• FiO2>50%
**Laboratory investigations**
• Hemoglobin < 7
• Platelets < 50
• INR out of therapeutic range
• Blood glucose < 4mmol/L or >20mmol/L
• Severe electrolyte derangements
**Neurological contraindications**
• Unstable ICP and exceeds 15mmHg in past 24 hours
• EVD output > 100ml in 24 hours; bloody EVD output; EVD lower than 10cmH2O above tragus of ear
**Orthopaedic contraindications**
• Spinal fractures, patient on spinal nursing, lower limb fractures awaiting fixation
**Vascular conditions**
• Untreated deep vein thrombosis /Pulmonary Embolism
• Severe limb ischemia/
• Active bleeding
• Any untreated aneurysm
• On intravenous Heparin
**Surgical issues or medical procedures**
• Attachments that contraindicate mobilisation (e.g., femoral sheaths, IABP, Swan-Ganz catheter, temporary pacing wire)
• Ordered Complete Rest In Bed due to medical issues/recent removal of invasive attachments
• Open abdomen
**Others**
• Body temperature > 39°C

• RASS score less than -1 or more than 2

MAP – Mean arterial pressure; Pa02 – Partial pressure of oxygen; Fio2 – Fraction of inspiratory oxygen concentration; PEEP – Positive end expiratory pressure; INR –International normalized ratio; ICP – intra cranial pressure; EVD – Extra ventricular drainage; RASS-Richmond agitation sedation score.
